# A Label-Free Microfiber Biosensor for Auxiliary Diagnosis of Pre-Eclampsia

**DOI:** 10.1021/acssensors.5c03162

**Published:** 2025-12-04

**Authors:** Zefeng Li, Danfeng Zeng, Yangjie Li, Yanliang Huang, Yi Zhou, Huijuan Quan, Yu Xie, Peishan Chen, Ruen Xie, Lan Rao, Xinzhu Sang, Gerald Farrell, Jinhui Yuan, Guoyong Sun, Qiang Wu

**Affiliations:** † State Key Laboratory of Information Photonics and Optical Communications, 12472 Beijing University of Posts and Telecommunications, Beijing 100876, China; ‡ Department of Gynaecology and Obstetrics, The Second Affiliated Hospital of Shantou University Medical College, Shantou University, Shantou 515041, China; § Department of Applied Physics, Science College, Shantou University, Shantou 515041, China; ∥ Radiology Department, 74599 The Second Affiliated Hospital of Shantou University Medical College, Shantou 515041, China; ⊥ Photonics Research Centre, School of Electrical and Electronic Engineering, City Campus, 8819 Technological University Dublin, Dublin 7, Ireland; # School of Engineering, Physics and Mathematics, 5995 Northumbria University, Newcastle upon Tyne NE1 8ST, U.K.

**Keywords:** cascade microfiber biosensor, vernier effect, pre-eclampsia, placental growth factor, auxiliary diagnosis

## Abstract

Pre-eclampsia (PE) is a serious multiorgan complication that can seriously threaten the life and health of pregnant women and their fetuses. Current clinical diagnosis relies heavily on nonspecific symptoms, while conventional biomarker assays lack the sensitivity to detect low concentrations of placental growth factor (PlGF), a key indicator whose levels drop significantly as PE progresses. This paper proposed a cascade microfiber (CMF) biosensor that utilizes the vernier effect for the quantitative detection of PlGF in clinal serum samples of PE patients. The experimental results show that the proposed CMF biosensor has a limit of detection as low as 0.49 pg/mL and a detection time that is less than 20 min. Clinical validation using serum samples from 35 pregnant women demonstrated that the CMF biosensor achieved 78.6% sensitivity, 85.7% specificity, and 82.9% diagnostic accuracy. Importantly, we have established a strong correlation between PlGF levels and clinical severity, confirming the biomarker’s auxiliary diagnosis value and reinforcing the sensor’s clinical relevance. The proposed method could form the basis of a next-generation diagnostic system for PE that combines high sensitivity, speed, and simplicity and has the potential to transform current screening protocols by enabling early intervention and improving maternal–fetal outcomes.

## Graphical abstract

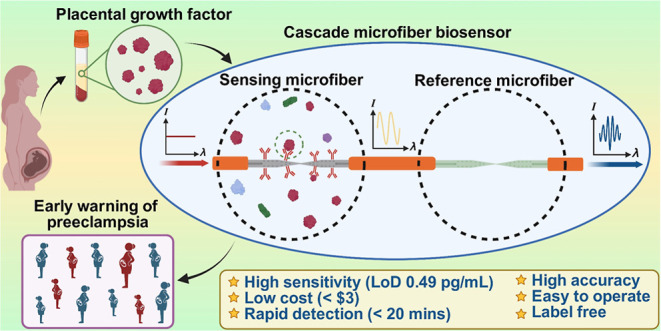

Preeclampsia (PE) is one of the leading causes of maternal and perinatal deaths worldwide. It is a severe form of pregnancy-induced hypertension. The proportion of pregnant women suffering from preeclampsia globally ranges from 2% to 8%. According to recent statistical data, approximately 70,000 pregnant women and over 500,000 perinatal infants die from the PE and related hypertensive diseases each year.^1–4^ PE can lead to adverse pregnancy outcomes and is a unique complication during pregnancy,^5^ which can endanger the lives of pregnant women and fetuses. Early detection of the onset of PE is vital to allow effective treatment. Therefore, finding a sensitive monitoring indicator is particularly important for early accurate diagnosis of PE. At present, the diagnosis and assessment of PE mainly rely on monitoring the blood pressure and detecting proteinuria, etc. The detection rate is relatively low, while the rate of missed diagnosis is relatively high. Relying on these nonspecific indicators to predict the occurrence of PE and to assess the prognosis of pregnant women and fetuses results in low sensitivity and specificity. It has been recognized that early detection of PE is not only key to reducing the incidence of PE and reverse adverse pregnancy outcomes but also vital as a contribution to better understanding the causes and mechanisms of PE.^6^

The placental growth factor (PlGF) is an important member of the vascular endothelial growth factor family and was first isolated and purified from a human placental genomic DNA libraries in 1991.^7^ The role of PlGF in the placenta is to promote angiogenesis, and its main function in nonplacental tissues is to respond to angiogenesis caused by pathological ischemia or injury.^8^ PlGF is a biomarker of angiogenesis, whose reduction may lead to placental vascular remodeling disorders, shallow placental implantation, and placental ischemia, thereby causing PE. Placental ischemia and hypoxia can lead to a reduced PlGF secretion, which in turn affects trophoblast cell migration, invasion, and spiral artery remodeling.^9^ Studies have shown that there are significant differences in PlGF levels between healthy pregnant women and patients with PE. The serum PlGF level for a PE patient is significantly lower than that of healthy pregnant women, and as the severity of the disease increases, the PlGF level in PE women gradually decreases.^10^ Typically, the level of PlGF in the serum of healthy pregnant women is in the range of several hundred to 1000 pg/mL.^11,12^ However, for some pregnant women with PE, the PlGF level can be extremely low. Meler et al. reported that PlGF levels in PE pregnant women were very low (<12 pg/mL). Such low PlGF concentrations pose a challenge, which in turn requires very low detection limits for PlGF biosensors.^13^ Moreover, studies have shown that the concentration level of PlGF in pregnant women is closely related to symptoms such as hypertension, proteinuria, and liver dysfunction.^14,15^

Currently, the methods used in clinical practice to detect PlGF include electrochemiluminescence immunoassays, fluorescence immunoassays, and enzyme-linked immunoassays.^16^ The main issues with these methods are their high cost and complex processing steps, which hinder their adoption in universal screening programs in hospitals.^17^ Recently, some new biosensors for PlGF detection have been proposed. In 2021, Pham et al. reported an electrical biosensor for detecting PlGF with a limit of detection (LoD) of 0.06 pg/mL within 40 min.^18^ In 2024, Soman et al. proposed a label-free electrochemical immunosensor for PlGF detection with a LoD of 53 pg/mL over a linear range from 1 to 1000 ng/mL.^19^ However, these sensors have clear disadvantages, such as a vulnerability to electromagnetic interference and complex manufacturing processes.^20–22^ At present, there is an urgent need to develop a simple, low-cost, and ultrasensitive biosensor to enhance the prediction of PE onset and facilitate timely monitoring of patient symptoms, ensuring optimal clinical decision-making and associated treatments.

With the development of biosensor technology, microfiber (MF) biosensors have attracted substantial attention as the basis of analysis and diagnostic tools, which can meet the needs of clinical testing for rapid, simple, selective, label-free, and low-cost analysis.^23–28^ A comprehensive review of MF sensor structures was summarized in ref 29. The vernier effect, which utilizes the interference between two optical modes with slightly different path lengths in order to magnify sensitivity, has recently proven to be a highly effective means for significantly improving the sensitivity of MF biosensors and thus enhancing their LoD.^30^ In the past few years, MF biosensors based on the vernier effect have been applied to detect a variety of biomarkers, including cytokeratin 19 fragment, AKT protein, progastrin-releasing peptide, etc.^31–33^ In addition, the rapid development of nanotechnology has enhanced the effectiveness of MF-based biosensors for disease detection due to their unique properties, such as large specific surface area, good chemical stability, and electrochemical properties. Carbon-based materials like carbon nanotubes and graphene oxide (GO) are typical of the type of nanomaterials that could be combined with the MF biosensors for medical biomolecular detection.^34–38^ The large specific surface area of such carbon-based materials and their own functional groups provide numerous reaction sites, enabling the nanomaterial to interact with a large number of biomolecules.^39–41^ The combination of MF biosensors based on the vernier effect, functionalized using carbon-based materials, provides a potential solution to detect ultralow concentrations of PlGF in the serum of PE patients.

In this paper, a vernier effect-based, high sensitivity cascade microfiber (CMF) biosensor is proposed and investigated for rapid, label-free detection of clinical serum samples. The sensing MF surface is immobilized with a carboxylated multiwalled carbon nanotube (CMWCNT)/GO/PlGF antibody layer, which will specifically bind with target PlGF in the analyte solution. The proposed CMF biosensor achieves a wide dynamic range and a low LoD in both PBS buffer and serum samples. Furthermore, comparison to results from a commercial enzyme-linked immunosorbent assay (ELISA) demonstrates the superiority of the CMF biosensor. In the testing of 35 clinical samples, the area under the receiver-operating-characteristics (ROC) curve (AUC) confirms PlGF’s clinical value as an auxiliary diagnostic biomarker and the feasibility of detecting PlGF using the proposed CMF biosensor. Based on the concentration levels of PlGF in the collected samples, the auxiliary diagnostic value of the concentration of PlGF and different PlGF cut-off values for different types of patients is studied.

## Materials and Methods

### Materials

Detailed information about the materials used in this paper is provided in the Supporting Information.

### Sensing Principle and Fabrication of the CMF Biosensor

Figure 1a shows a schematic diagram of the pathophysiology and characteristics of PE. As can be seen from Figure 1a, the agonistic angiotensin II type-1 receptor autoantibodies (AT1-AAs), natural killer cells (NK cells), oxidative stress, and genetic factors can all affect the concentration changes in PlGF in the placenta. PE is associated with abnormal PlGF concentration level caused by placental dysfunction, which can lead to a series of adverse maternal outcomes, such as hypertension, proteinuria, HELLP syndrome, placental abruption, liver dysfunction, preterm birth or stillbirth, renal injury, and growth restriction.^14,15,45^

**Figure 1:**
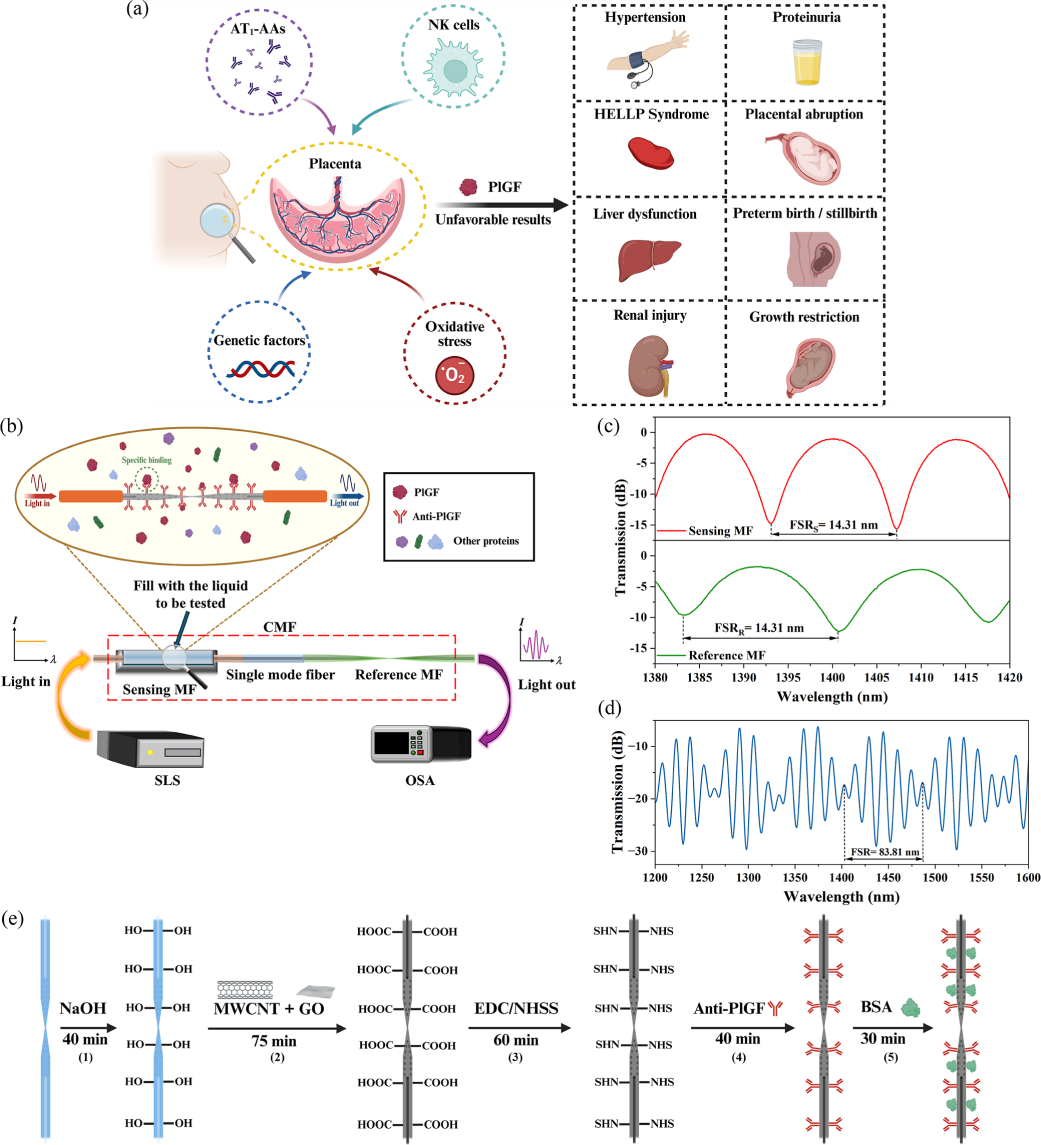
(a) Schematic diagram summarizing the pathophysiology and features of PE. (b) The schematic configuration of the proposed CMF biosensor. (c) The transmission spectra of the sensing MF, reference MF, and (d) CMF. (e) The steps involved in modifying the sensing MF surface.

Figure 1b shows a schematic diagram of the CMF sensing system, which includes a supercontinuum source (SLS, Anyang, SC-5), two MFs (one acts as the sensing MF, and the other acts as a reference MF), and a high-resolution optical spectrum analyzer (OSA, AQ63370C, YOKOGWA). The interference spectra of the two cascaded MFs form a superposition spectrum, which allows the vernier effect to be used. The sensing MF is immersed into the PlGF analyte, and the PlGF antibody on the sensing MF will specifically bind with the target PlGF protein, causing the effective thickness change of the CMWCNTs/GO film, resulting in a wavelength shift of the output spectrum. The detailed fabrication process and experimental setup of the CMF biosensor are shown in Supporting Information. A numerical simulation study of the CMF biosensor was undertaken using the finite element method and beam propagation method, and the simulated field distributions of the supermodes and light transmission can be found in the Supporting Information.

The output spectrum of the sensing MF and the reference MF is shown in Figure 1c. When the sensing MF and the reference MF are cascaded, forming a CMF, the resultant output spectrum is shown in Figure 1d. The corresponding detailed description of Figure 1c and Figure 1d can be found in the Supporting Information.

Figure 1e shows a schematic diagram of the sensing MF surface modification processes. The corresponding detailed description can be found in the Supporting Information.

## Results and Discussion

### Characterizations of the CMF Biosensor

The refractive index (RI) sensitivity of the proposed CMF was experimentally investigated. The experiment first measured the RI range of 20 anonymous serum samples, which were diluted 5-fold, the same dilution factor used in subsequent tests. The RI values were mainly distributed between 1.3344 and 1.3375, with a median of 1.337. As shown in Figure 2a, the RI distribution is “violin-shaped”. Figure 2b to Figure 2e show the experimental results of the CMF sensor’s RI and temperature sensitivity. Detailed descriptions can be found in the Supporting Information.

**Figure 2:**
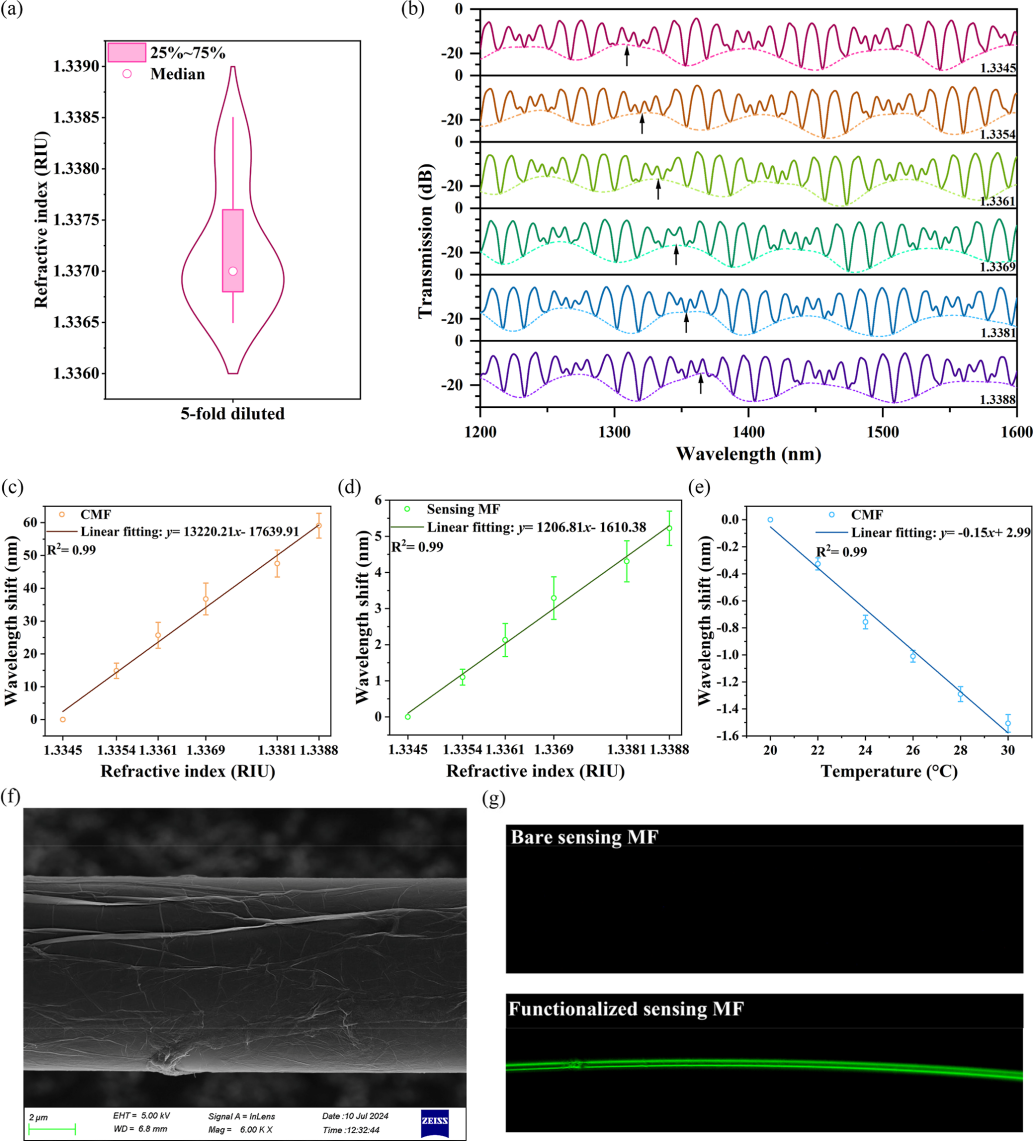
(a) RI distribution of the serum diluted 5-fold. (b) Measured transmission spectral response of the CMF under different RI conditions. RI sensitivities of the (c) CMF and (d) sensing MF alone. (e) Temperature sensitivity of the CMF. (f) The SEM image of the sensing MF surface after modification of CMWCNTs/GO. (g) The images from fluorescence micrographs of the bare sensing MF and PlGF antibody-functionalized sensing MF.

Figure S2 shows the characterizations of the CMF biosensor determined by X-ray photoelectron spectroscopy, indicating that the CMWCNTs/GO film successfully adhered to the sensing MF surface and that 1-(3-(Dimethylamino)­propyl)-3-ethylcarbodiimide hydrochloride (EDC)/N-27 Hydroxysuccinimide (NHS) as attached to the CMWCNTs/GO film. The detailed description of Figure S2 can be found in the Supporting Information. Figure 2f shows the scanning electron microscopy (SEM) image of the sensing MF surface immobilized with CMWCNTs and GO, which verifies that the CMWCNTs/GO film successfully adhered to the sensing MF surface. Figure 2g shows the fluorescence micrograph of the surface of bare sensing MF and functionalized sensing MF, indicating that the PlGF antigen protein has been successfully immobilized onto the sensing MF surface. The detailed description of Figure 2g can be found in Supporting Information.

The sensing MF optical data for the deposition process associated with steps (1) and (2) and steps (4) and (5) in Figure 1e are shown in Figure S3a and Figure S3b in the Supporting Information, respectively.

### Characterization of the CMF Biosensor to PlGF in PBS

Before undertaking PlGF detection, the fabrication repeatability and short-term output wavelength stability of the CMF biosensor was studied. Figure 3a shows the test results of these five CMF biosensors, indicating that the developed CMF biosensors demonstrate good repeatability. In addition, the results also demonstrate that each of the CMF biosensors possesses good wavelength stability with time, and a detailed description can be found in the Supporting Information.

**Figure 3:**
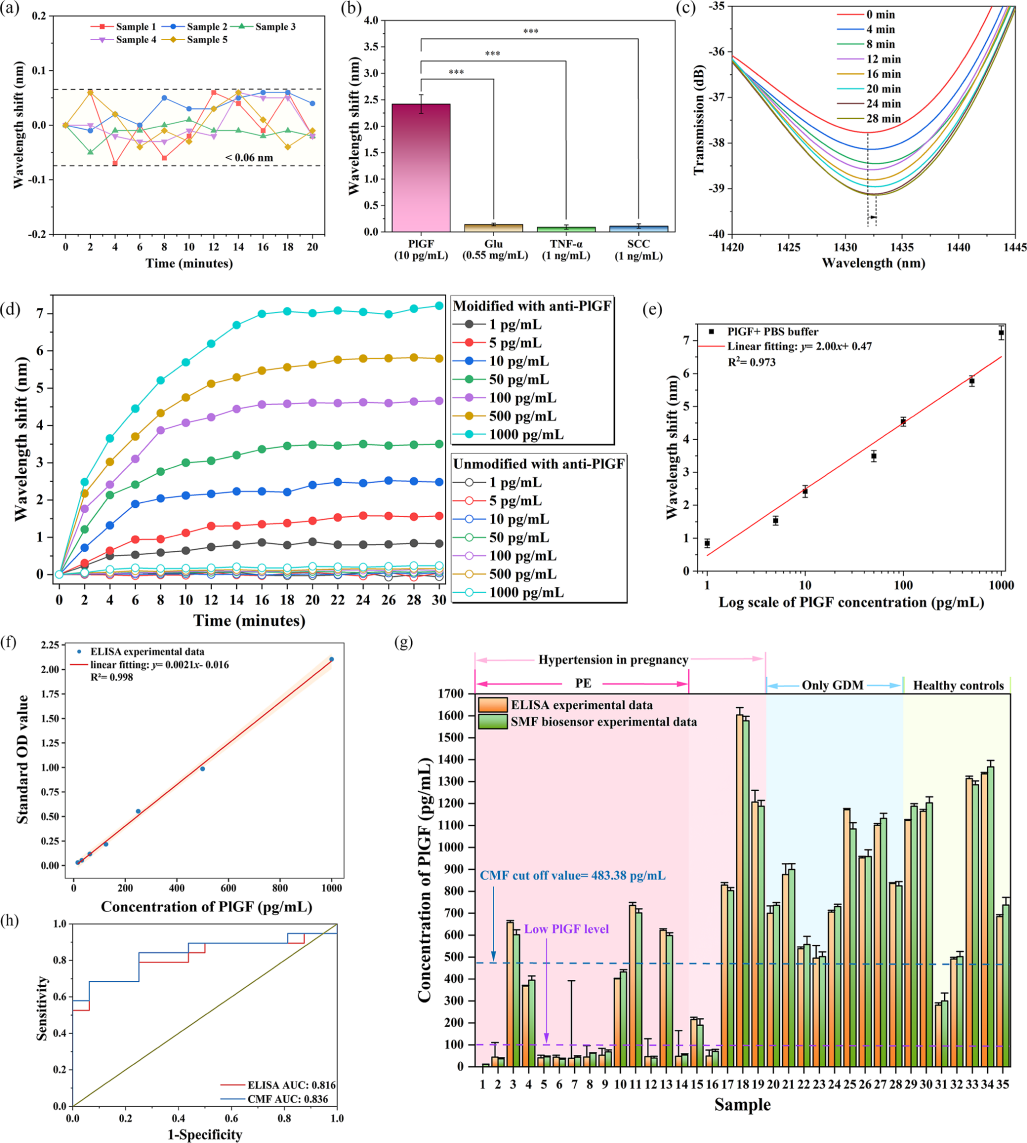
(a) Repeatability and stability measurements for the CMF biosensor samples in PBS buffer. (b) Specificity test results of the CMF biosensors. (c) The dynamic shift of the CMF biosensor in 1 pg/mL. (d) The envelope wavelength shift of the CMF biosensors in different concentrations of PlGF in PBS buffer. (e) PlGF sensitivity of the CMF biosensor in PBS buffer. (f) The PlGF sensitivity of ELISA. (g) The PlGF concentrations obtained from serum samples were assayed by the ELISA and CMF biosensors, respectively. (h) The AUC curves of the ELISA and CMF biosensors.

The specificity of a biosensor is a crucial parameter in real-world applications, and for this reason, specificity tests were also initially carried out. The specificity tests for the CMF biosensor were undertaken by immersing the CMF in four different types of solutions: PlGF, glucose (Glu), tumor necrosis factor-α (TNF-α), and squamous cell carcinoma (SCC). Figure 3b shows the specificity test results for the CMF biosensor, indicating that the proposed CMF biosensor has high specificity. The reason for selecting these reference solutions and the detailed description of the specificity testing can be found in the Supporting Information.

To enable the sensor to function within the clinically valuable range of PlGF concentrations, we referred to previous meta-analysis reports on the PlGF levels in the serum of pregnant women with PE. Based on this, we determined the necessary testing range of the CMF biosensor should be 1–1000 pg/mL. Figure 3c shows the interference envelope spectra of the CMF biosensor vs time when it was immersed into PlGF solution with a concentration of 1 pg/mL. The dip wavelength of the interference envelope spectra experiences a monotonic red-shift as time increases and remains unchanged after 20 min. The detection time therefore is determined to be no more than 20 min. Figure 3d illustrates the envelope wavelength shift of the CMF biosensors at different concentrations of PlGF. The envelope wavelength shift increases as the concentration of PlGF increases. It is important to note also that for the unfunctionalized CMF, as the concentration of PlGF increases, there is no apparent dip wavelength shift over time, because PlGF cannot bind onto the CMF sensor, due to absence of functionalization of the sensing MF. Figure 3e shows the linear fitting results (*n*= 3) between the envelope wavelength shift and log scale of the concentration of the PlGF in PBS buffer, with the LoD of 0.49 pg/mL. The detailed description of Figure 4e and LoD calculation can be found in the Supporting Information. In addition, given the linearity of the sensors response, the dynamic range of the CMF biosensor can be determined to be at least equal to the testing range. The dynamic range of the CMF biosensor is therefore determined to be 1 pg/mL and 1 ng/mL.

**Figure 4:**
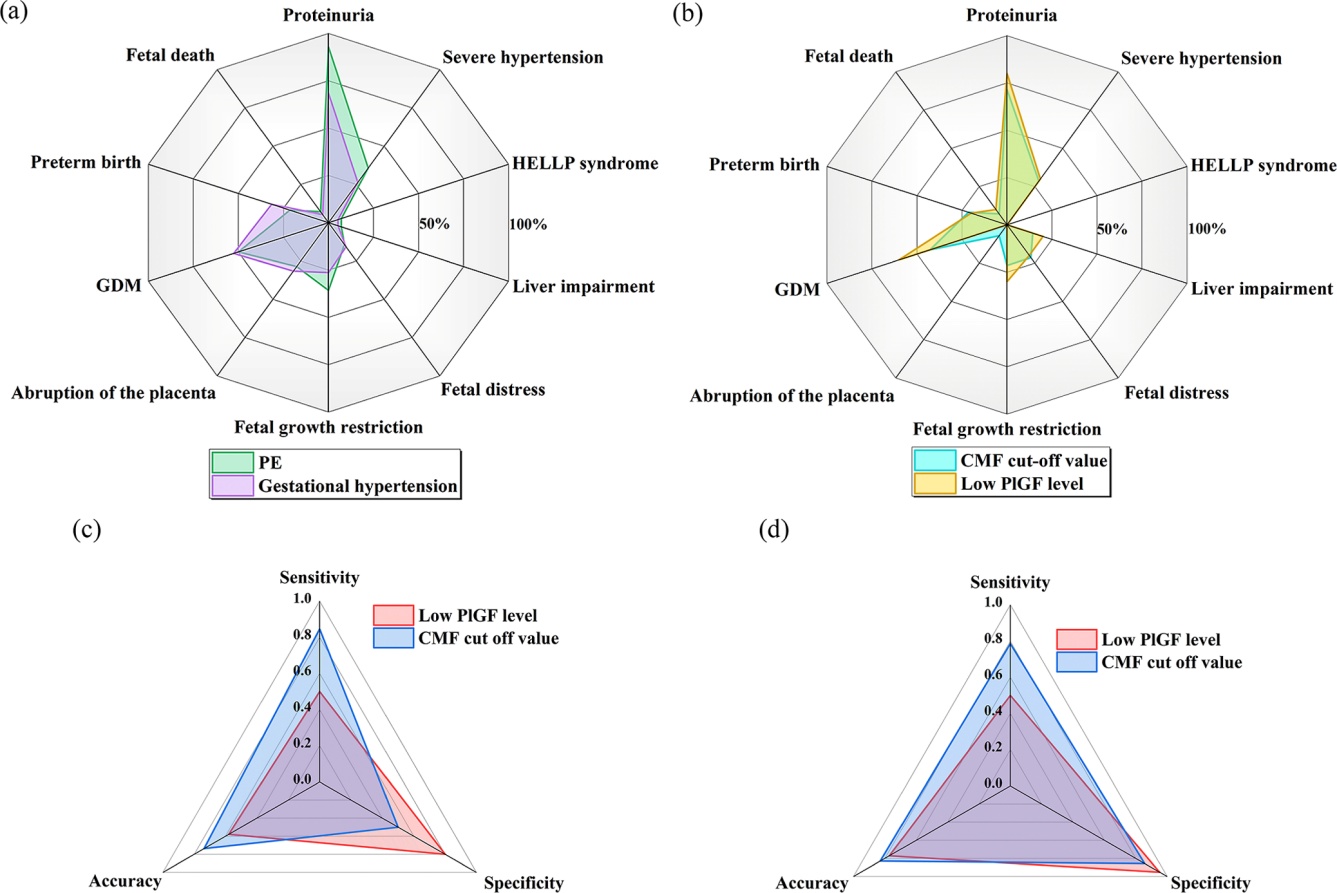
(a) Statistics on selected clinical symptoms in patients with PE and patients with gestational hypertension. (b) Statistics of selected clinical symptoms of patients screened using CMF cut-off value and low PlGF level, respectively. The screening performance of the CMF cut-off value method and the low PlGF level method in (c) the patients with gestational hypertension and (d) all collected patients.

Table S1 compares the results of the proposed CMF biosensor with those of other reported or commercialized methods for PlGF detection. The proposed CMF biosensor has the advantages of low cost, rapid detection, high sensitivity, and low LoD, which confirms its potential to be widely used in the rapid screening and detection of PE patients and the study of the predictive values of PlGF, and detailed descriptions can be found in the Supporting Information.

### Detection of PlGF in Clinical Samples

After successful characterization of the CMF biosensor for PlGF detection in a PBS sample, the sensor was used for PlGF detection in clinical samples. The samples were obtained and approved from The Second Affiliated Hospital of Shantou University Medical College (Approval Number: 2022–101). The use of human serum samples in this study was approved by The Ethics Department of the Second Affiliated Hospital of Shantou University Medical College. Samples were made available from 35 pregnant women, and baseline characteristics of all collected pregnant women and the diagnostic information on PE can be found in the Supporting Information. To verify the accuracy of the results using the CMF biosensor, ELISA (MULTI SCIENCES PlGF ELISA Kit) was used to test the same patient samples, and Figure 3f shows the linear fitting results of the ELISA Kit. The assay procedure utilizing ELISA can be found in the Supporting Information.

For clinical serum samples tested with the CMF biosensor, to eliminate errors caused by nonspecific binding and nonspecific precipitation, all the serum samples were diluted five times with PBS before testing, and each diluted sample was tested two times using a different CMF biosensor. Figure 3g shows the tested results by ELISA and CMF. The experimental results show that ELISA and CMF biosensors have similar tested PlGF values for the same serum samples (except sample 1 where ELISA does not have a test value), which demonstrates that the proposed CMF biosensors could be used for reliable detection in clinical samples. As shown in Figure 3h, the ROC indicated that the serum PlGF level in the clinical samples was a suitable classification index for distinguishing PE patients from gestational diabetes mellitus (GDM) patients or healthy people. The AUCs of the ELISA and CMF biosensors were 0.816 and 0.836, respectively, indicating that the discrimination performance of the CMF biosensor is slightly better than that of ELISA. The cut-off value of the CMF biosensor can be calculated by Youden’s index and AUC. Youden’s index can be expressed as

1 $$Youden^{\prime }s\;index=sensitivity+specificity-1$$

and the cut-off value is obtained by calculating the PlGF concentration corresponding to the maximum value of Youden’s index, which is 483.38 pg/mL.

### The Auxiliary Diagnosis Value of PlGF and Screening Performance of the CMF Biosensors for PE

To evaluate the auxiliary diagnostic value of PlGF, we assessed the clinical severity of the PE patients including fetal death, preterm birth, GDM, abruption of the placenta, fetal growth restriction, fetal distress, liver impairment, HELLP syndrome, severe hypertension, and proteinuria, as shown in Figure 4a. The detailed description of Figure 4a can be found in the Supporting Information. The percentage of adverse symptoms was higher in patients with PE than in patients with gestational hypertension. Adverse outcomes for pregnant women and fetuses in patients with PE are primarily characterized by renal impairment suggestive of proteinuria, severe hypertension, fetal growth restriction, and GDM.

The serious adverse outcomes mentioned above for pregnant women and fetuses suggest that early screening for PE and rapid clinical diagnosis are of great value. Several studies have shown that determining if PlGF is at low levels (<100 pg/mL) is an effective means to identify patients at risk from PE and hypertension in pregnancy.^46,47^ To examine this further, we identified two distinct scenarios: one based on the cut-off value of PlGF detected by the CMF biosensor (483.38 pg/mL in Figure 3g), and the other based on a low level of PlGF. Figure 4b shows the severe symptoms suffered by patients screened by the CMF cut-off value and low PlGF level, respectively. The detailed description of Figure 4b can be found in the Supporting Information. The percentage of patients auxiliary diagnosed and screened with clinically severe symptoms using the low PlGF level method was significantly higher than that using the CMF cut-off value. This means that it is more effective to screen patients with severe clinical symptoms using a low PlGF level because PlGF level decreases as PE worsens.

Figure 4c and Figure 4d shows the screening effect of the low PlGF level method and the CMF cut-off value method on all patients with pregnancy-included hypertension, respectively. The detailed description of Figure 4c and Figure 4d can be found in the Supporting Information. Based on the sample size of this research, the accuracy of the CMF cut-off value method was slightly higher than that of the low PlGF level method. While the sensitivity of the CMF cut-off value method is higher, it is worth noting that the specificity of the low PlGF level method is higher.

It should be noted that due to the variations in the sample sizes reported by other research groups, especially in reports with smaller sample sizes, the cut-off value may exhibit some fluctuations. However, it is believed that this work can provide a new approach for the auxiliary diagnosis of PE patients. The CMF biosensor can serve as a full-term screening tool for PE using serum biomarkers. Based on the current sample size, we can make a reasonable inference that the CMF biosensor enables prompt assessment of PE risk in pregnant women, assists doctors in identifying high-risk patients, enhances monitoring and proactive intervention, and facilitates early PE detection, thereby reducing the likelihood of adverse pregnancy outcomes. For large-scale screening, the high sensitivity of the CMF cut-off value method enables timely screening of potential patients, thereby avoiding missed diagnoses. For patients with severe symptoms who require close follow-up, the high specificity of the low PlGF level method is a better choice, and the low levels of PlGF can serve as an auxiliary diagnostic indicator to help doctors avoid misdiagnosis. Moreover, the PlGF level decreases with the severity of the disease, and the PlGF concentrations may be so low that they cannot be detected by commercially available PlGF assays. In this case, the advantages of the proposed CMF biosensor are more pronounced.

## Conclusion

In summary, a novel CMF biosensor based on the vernier effect is proposed to detect PlGF in clinical serum samples. The EDC/NHS-activated CMWCNTs/GO films were selected to chemically immobilize PlGF antibodies onto the MF sensor surface and thus enrich the binding sites for immobilizing a larger quantity of anti-PlGFs onto the MF sensor surface. The experimental results show that the proposed CMF biosensor has a LoD of 0.49 pg/mL over the PlGF concentration range from 1 pg/mL to 1 ng/mL, and the detection time is less than 20 min. With a total of 35 patients and controls, the CMF biosensor detects PE with an AUC of 0.836. We compared the detection results of CMF with those of commercial ELISA kits. The coefficient value was less than 10%, which proved the reliability of the detection results of the CMF biosensor. Furthermore, based on the sample size of this study, we analyzed the sensitivity, accuracy, and specificity of using the low PlGF value and CMF cut-off value method in the entire sample as well as in patients with only gestational hypertension. Among patients with only gestational hypertension, the low PlGF level has higher specificity and is suitable for auxiliary diagnosis. Among all the samples we have collected, the CMF cut-off value has a higher sensitivity and is more suitable for large-scale screening. In the future, the proposed CMF biosensor can provide a promising method for early detection and auxiliary diagnosis of PE in a clinical environment.

## Supplementary Material



## Notes

Materials; fabrication and sensing principle of the cascade microfiber biosensor; refractive index and temperature sensitivity of the CMF biosensor; functionalization process of the CMF biosensor; repeatability, stability, and specificity of the CMF biosensor; comparison of detection performance of different PlGF sensors; the detection process of ELISA; the diagnostic basis of PE and the classification of clinical samples; and the auxiliary diagnosis value of PlGF (PDF)
