# Supplementary material for: A Label-Free Microfiber Biosensor for Auxiliary Diagnosis of Pre-Eclampsia
Source: ACS Sens. 2025 Dec 4;11(1):468–77. doi: 10.1021/acssensors.5c03162 (PMC12836341; doi:10.1021/acssensors.5c03162)
Supplement: Supplementary file 1 [file se5c03162_si_001.pdf]

Supporting Information

A label-free microfiber biosensor for auxiliary diagnosis of pre-eclampsia

Zefeng Li[1,‡], Danfeng Zeng[1,‡], Yangjie Li[3], Yanliang Huang[3], Yi Zhou[3], Huijuan Quan[3], Yu Xie[3], Peishan Chen[2], Ruen Xie[3], Lan Rao[1], Xinzhu Sang[1], Gerald Farrell[5], Jinhui Yuan[1,], Guoyong Sun[3,*], and Qiang Wu[6,*]*

[1]State Key Laboratory of Information Photonics and Optical Communications, Beijing University of Posts and Telecommunications, Beijing, 100876, China

[2]Department of Gynaecology and Obstetrics, The Second Affiliated Hospital of Shantou University Medical College, Shantou University, Shantou, 515041, China

[3]Department of Applied Physics, Science College, Shantou University, Shantou, 515041, China

[4]Radiology Department, The Second Affiliated Hospital of Shantou University Medical College, Shantou University, Shantou, 515041, China

[5]Photonics Research Centre, School of Electrical and Electronic Engineering, City Campus, Technological University Dublin, Dublin 7, Ireland

[6]School of Engineering, Physics and Mathematics, Northumbria University, Newcastle upon Tyne, NE1 8ST, United Kingdom

Corresponding Authors: yuanjinhui81@bupt.edu.cn; *gysunup@stu.edu.cn; qiang.wu@northumbria.ac.uk*

‡These authors contributed equally.

Materials

The seven-core fiber was purchased from FIBERCORE Co.. The single mode fiber was purchased from Yangtze Optical Fibre Co.. The placental growth factor (PlGF), anti-PlGF, and fluorescent protein were purchased from OriGene Technologies, Inc. The graphene oxide (GO) and carboxylated multi-walled carbon nanotubes (CMWCNTs) were purchased from Nanjing XFNANO Materials Tech Co. Ltd.. N-27 Hydroxysuccinimide (NHS), 1-(3-Dimethylaminopropyl)-3-ethylcarbodiimide hydrochloride (EDC), Bovine serum albumin (BSA) and phosphate buffered solution (PBS) were purchased from Shanghai Yuanye Biotechnology Co. Sodium chloride (NaCl) and glucose powder were purchased from Fuchen Chemical Reagent Co. The PlGF ELISA kit was purchased from Multisciences (Lianke) Biotech, Co., Ltd.. Tumor necrosis factor-α and squamous cell carcinoma were purchased from Shanghai Linc-Bio Science Co. Ltd..

Fabrication and Sensing Principle of the Cascade Microfiber Biosensor

The microfiber (MF) is fabricated by the flame-heating method. A short section of 1 cm seven core fiber is fusion-spliced between two single model fibers by a commercial fusion splicer (FSM-61S, Fujikura). As showed in **Figure S1(a)**, the fiber flame-heating system consists of a hydrogen flame torch, two motorized translation stages, and a computer. The optical fiber is fixed on fiber clamps and preheated for 3.6 seconds before being stretched by a pair of high-precision translation stages controlled by the computer. During the tapering process, the hydrogen flow rate, oxygen flow rate, and drawing speed are optimized to 70 mL/min, 20 mL/min, and 65 μm/s, respectively. The flame is scanned back and forth with the amplitude gradually changing from 1200 to 3600 μm, and the stretch

length and the taper diameter of the sensing MF and reference MF are 12500 and 11000 μm, and 8.5 and 10.5 μm, respectively. The structure parameters and details of the seven core fiber can be found in Reference [1]. Specifically, the diameter of the core and core spacing of the seven core fiber are 6.1 and 35 μm, respectively.

The mode distributions and energy distribution characteristics of the tapered seven core fiber are investigated using COMSOL Multiphysics. **Figure S1(b)** shows the four typical mode distributions of the seven core fiber by the finite element method, and the waist diameter of the fiber is set as 10 μm. **Figure S1(c)** shows the energy distribution along the transmission distance by the beam propagation method. As the diameter of the waist diameter decreases, the ability of the central core to limit the mode field decreases, and the mode field begins to spread outwards and interact with the surrounding core, resulting in supermodes interference. The energy in the central core decreases rapidly as the light passes through the waist region, and the supermodes interference is clearly observed. P_1 and P_2 represent the energy of the center core and the surrounding core, respectively. When the light passes the non-taper region, the energy can be well confined in the center core. When the light passes the taper region, the periodic energy occurs to change along the tapered section.

The cascade microfiber (CMF) biosensor spectrum is the product of the reference and sensing spectra and can be described by the following equation [2-4]:

$$I_{CTSCF} = I_r \times I_s = A_r A_s + A_r B_s \cos\left(\frac{2\pi}{\lambda}\Delta n_{eff} L_s\right) + A_s B_r \cos\left(\frac{2\pi}{\lambda}\Delta n_{eff} L_r\right) + \frac{1}{2} B_r B_s \cos\left[\frac{2\pi}{\lambda}\Delta n_{eff}(L_s + L_r)\right] + \frac{1}{2} B_r B_s \cos\left[\frac{2\pi}{\lambda}\Delta n_{eff}(L_s - L_r)\right], \quad (1)$$

where A_r and A_s are equal to the corresponding $I_{\text{core}} + I_{\text{clad}}$ of the reference MF and sensing MF, respectively. B_r and B_s are equal to $2\sqrt{I_{\text{core}} \cdot I_{\text{clad}}}$ for the corresponding reference MF and sensing MF,

respectively. Free spectral range (FSR) is an important parameter when characterizing optical interference, representing the wavelength interval between the adjacent spectral peaks or dips. The FSR for the reference free spectral ranges (FSR_R) and the sensing free spectral ranges (FSR_S) were 14.31 nm and 17.39 nm, as shown in **Figure 1(c)**. The FSR of the envelope (FSR_E) can be described as

$$FSR_{\mathrm{E}} = \frac{FSR_{\mathrm{S}} FSR_{\mathrm{R}}}{|FSR_{\mathrm{S}} - FSR_{\mathrm{R}}|}, \quad (2)$$

The FSR_E of the CMF is calculated to be 80.8 nm, which agrees well with the measured value of 83.81 nm, as shown in **Figure 1(d)**. According to Reference [2], the vernier effect envelope sensitivity S_C of the CMF can be described as

$$S_{\mathrm{C}} = \frac{FSR_{\mathrm{E}}}{FSR_{\mathrm{s}}} S_{\mathrm{s}}, \quad (3)$$

where S_s is the sensitivity of the sensing MF. As expected given the values of FSR_E and FSR_S the sensitivity $S_C > S_s$.

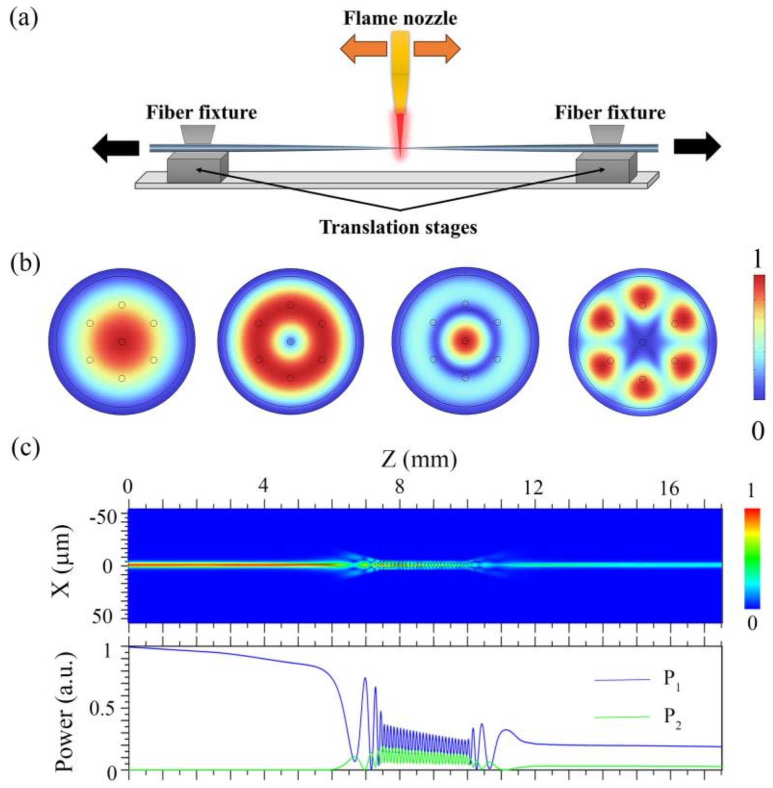

Figure S1. (a) Schematic diagram of the flame-heating method. (b) The simulated field distributions of the supermodes. (c) The simulated transmission optical field and energy distribution along X-Z direction of the sensing MF, where P_1 and P_2 are the energy of the center and surrounding core, respectively.

Refractive Index and Temperature Sensitivity of the CMF Biosensor

Figure 2(b) shows that the interference envelope spectra of the proposed CMF biosensor shifts towards longer wavelength as the RI increases from 1.3345 to 1.3388. **Figure 2(c)** and **Figure 2(d)** show that the RI sensitivities of the CMF (n= 3) and sensing MF (n= 3) are 13220.21 nm/RIU and 1206.81 nm/RIU, respectively, with corresponding factor R^2 values of 0.99. Therefore, the RI sensitivity of the CMF is 10.95 times higher than that of the sensing MF alone. The temperature sensitivity of the CMF (n= 3) is also investigated to study how much crosstalk is caused by changes in

ambient temperature. **Figure 2(e)** shows that the temperature sensitivity of the CMF is -0.15 nm/°C when the ambient temperature increases from 20 to 25 °C. In practice in our experimental procedure, the temperature is maintained at 25 °C ± 0.5 °C. As a result, the effect of ambient temperature change on the interference envelope spectra is very small compared to the effect of RI changes. Furthermore, since both the RI and temperature sensitivities exhibit an excellent linear response, the wavelength crosstalk caused by temperature fluctuation could be compensated for by using an additional temperature sensor.

Functionalization Process of the CMF Biosensor

All reagents were prepared from analytically pure reagents and sterilized deionized (DI) water. Before modification processes, the sensing MF was soaked in a 5% HNO_3 solution for 30 minutes to clean the sensing MF surface.

(1) The sensing MFs were immersed in a 5% NaOH solution for 40 minutes to generate sufficient silanol (Si-OH) groups on their surface. Subsequently, the MFs were thoroughly rinsed with deionized (DI) water and dried in an oven at 50 °C for 10 minutes.

(2) A mixed aqueous dispersion of carboxylated multi-walled carbon nanotubes (CMWCNTs) and graphene oxide (GO) at 2 mg/mL was sonically agitated for 30 minutes. The pre-treated MFs were then immersed in this dispersion for 75 minutes, allowing CMWCNTs and GO to covalently graft onto the MF surface via interactions between silanol and carboxyl (-COOH) groups. After modification, the MFs were sequentially washed with pure ethanol and then DI water, followed by drying at 50 °C for 10 minutes.

(3) The functionalized MFs were incubated in a solution containing 0.03 M EDC and 0.05 M NHSS in 0.01 M PBS (pH 7.4) for 1 hour at 25 °C to activate the carboxyl groups. The MFs were then rinsed with 0.01 M PBS buffer.

(4) The activated MFs were immersed in a 100 ng/mL solution of PlGF antibody for 40 minutes, facilitating the formation of amide bonds between the NHS-ester groups on the CMWCNTs/GO film and the C-terminus of the antibodies. After immobilization, the MFs were washed with 0.01 M PBS.

(5) Finally, the MFs were treated with 2% BSA solution in 0.01 M PBS for 30 minutes to block non-specific binding sites and remove excess antibodies, followed by a final wash with PBS.

Figure S2(a) shows the X-ray photoelectron spectroscopy (XPS, Thermo Kaplha, Thermo) spectrum of the sensing MF without and with EDC/NHSS modification, respectively. As shown in **Figure S2(a)**, the XPS spectrum without the EDC/NHS treatment shows C 1s and O 1s, and that with the EDC/NHS treatment shows C 1s, O 1s, and N 1s. After step ii, the XPS of C 1s has two peaks at C-C, C-O-C, C-OH, and O-C=O, respectively, as demonstrated in **Figure S2 (b)** and **Figure S2 (c)**. The O 1s spectrum can fit to be four peaks, which correspond to O-C=O, C=O, C-OH, and C-O-C, as shown in **Figure S2(d)** and **Figure S2(e)**. By immersing -COOH from CMWCNTs and GO to react with EDC/NHS, the N 1s core level peaks of the EDC/NHS activated sensor appear at 399.8, 401.6, and 402.6 eV, these peaks corresponding to the C-N, -CO-NH-, and C-NH^{3+}, respectively, as shown in **Figure S2(f)**. The changes of the N 1s XPS spectrum could be explained by the covalent linkage between CMWCNTs/GO film and EDC/NHS [5, 6].

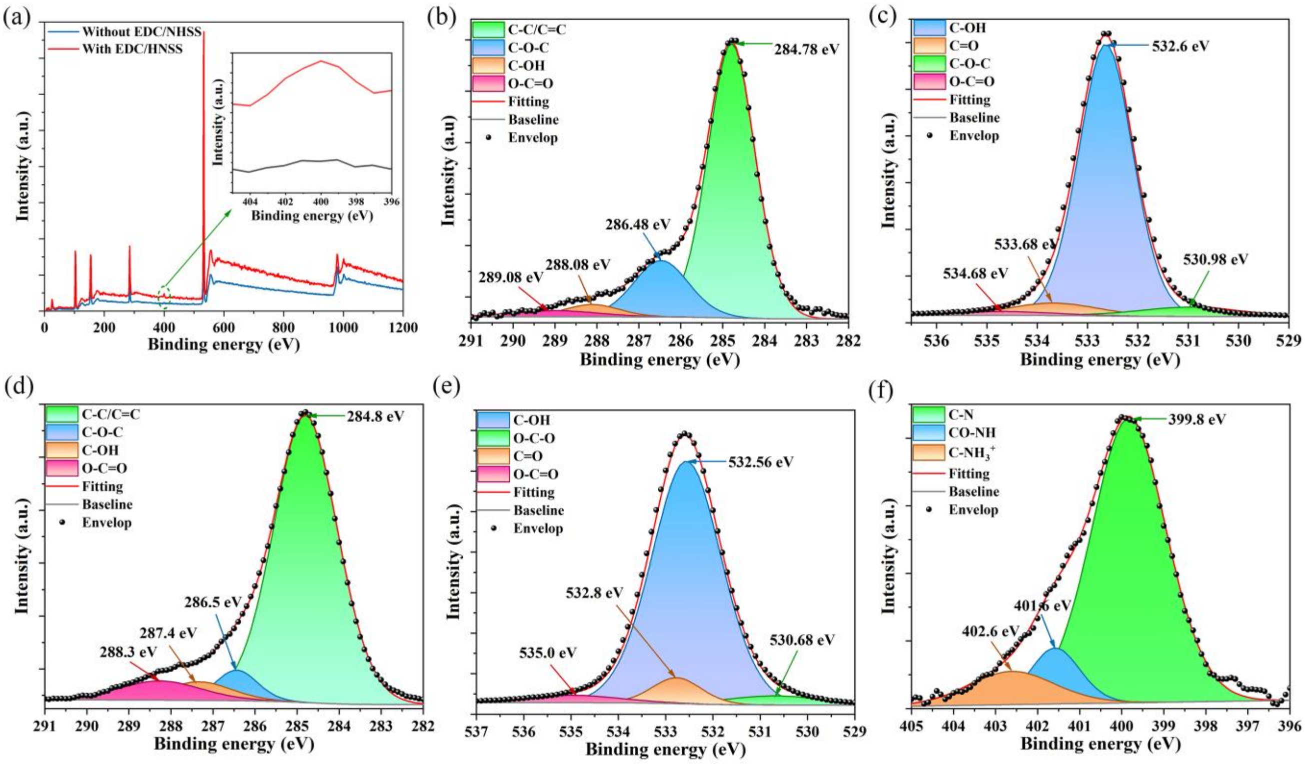

Figure S2. (a) The XPS spectrum of the sensing MF surface with/without EDC/NHS treatment. (b) C 1s and (c) O 1s XPS spectrum of the sensing MF surface without EDC/NHS treatment. The (d) C 1s, (e) O 1s and (f) N 1s XPS spectrum of the sensing MF surface with EDC/NHS treatment.

Figure 2(f) shows the scanning electron microscope image (SEM, Gemini 300, Zeiss) image of the sensing MF surface. Due to the presence of GO, the prepared mixtures show a corrugated texture morphologically, and GO covers almost the entire area due to the higher hydrophobicity of CMWCNTs than GO. In addition, the porous structure of the hybrid significantly improves the specific surface area, and 3-D spatial configuration formed via the supporting CMWCNTs/GO films enables more proteins to be absorbed effectively. The change in surface RI caused by protein adsorption to the film surface can be converted into a significant change in dielectric constant. In addition, there are studies indicating that modifying with two-dimensional material films can also contribute to the enhancement of the RI sensitivity [7-9].

To validate whether PlGF antibodies have been immobilized onto the sensing MF surface, the functionalized sensing MF was immersed in a fluorescent protein solution for 30 minutes. The fluorescent protein chosen can be specifically captured by the PlGF antibodies on the sensing MF, and observed using the fluorescence microscopy (Sainwoo Optical, ICX41, China). As shown in **Figure 3(g)**, the functionalized sensing MF surface showed a brighter green light compared with the image of the bare sensing MF, which confirms that the PlGF antigen has been attached onto the sensing MF surface, indicating that the PlGF antigen protein has been successfully immobilized onto the sensing MF surface.

As shown in **Figure S3(a)**, when the sensing MF is immersed in NaOH solution for surface hydroxylation treatment, the wavelength shifts towards shorter wavelengths and gradually stabilizes after 20 minutes. During the adsorption process of the CMWCNTs/GO film, the wavelength significantly shifts towards longer wavelengths in the first 10 minutes, and then slowly red-shifts as the immersion time increases. As shown in **Figure S3(b)**, when anti-PlGF is adsorbed onto the surface of sensing MF, the wavelength exhibits a significant redshift within the first 5 minutes, followed by a slow red-shifts from 5 to 25 minutes, and then stabilizes after 30 minutes. During the BSA sealing process, the wavelength shifts towards longer wavelengths within the first 20 minutes, and then stabilizes after the next 20 minutes.

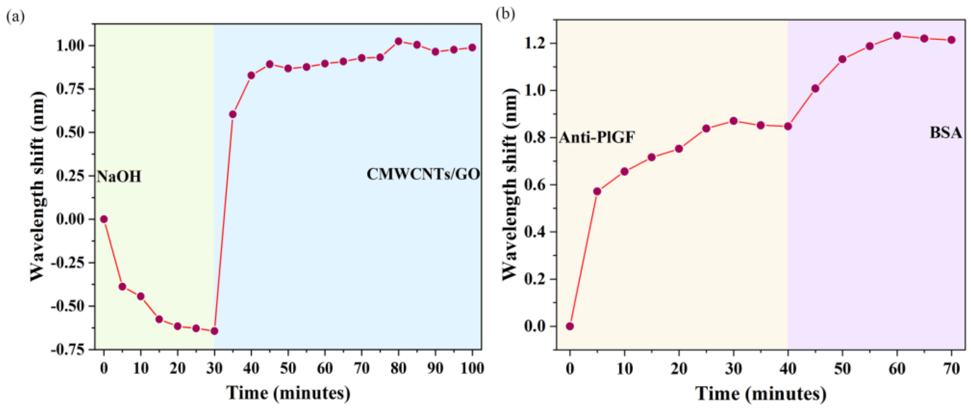

Figure S3. Dynamic spectrum of the functionalized sensing MF surfaces, corresponding to (a) the changes between step (1) and step (2), and (b) between step (4) and step (5), respectively.

Repeatability, Stability, and Specificity of CMF Biosensor

For repeatability and output wavelength stability of the CMF biosensor, five CMF biosensor samples were fabricated with the same sensor structure and functionalization parameters. All the CMF biosensor samples were immersed in a PBS buffer for 20 minutes. **Figure 3(a)** shows the test results for the five CMF biosensors. It can be seen from **Figure 3(a)** that the wavelength variations of five CMF biosensors are smaller than 0.06 nm over a period of 20 minutes.

The reason for the choice of glucose, TNF-α and SCC as interference biomarkers is because that patients with preeclampsia usually have GDM and may have some inflammatory response, and glucose and TNF-α are closely related to GDM and inflammation [10, 11]. Furthermore, PlGF is also related to cancers such as ovarian cancer or breast cancer, and SCC is one of the cancer markers [12, 13]. For the concentration of Glu, we selected three times the normal fasting blood Glu level of healthy individuals (<5.1 mmol/L) diluted by a factor of 5, as the Glu concentration value for measurements. For biomarkers, a concentration approximately ten times higher than the lower PlGF level was selected to validate the specificity of the CMF biosensor. As shown in **Figure 3(b)**, the value $^{*}P < 0.05$ indicates that the test was significant for the given sample group (t-Student test, $^{**}P < 0.01$, $^{***}P < 0.001$, $^{****}P < 0.0001$), the detection response of PlGF is significantly greater than that of the control samples, indicating that the proposed CMF biosensor has a high specificity.

The linear fitting in **Figure 3(e)** is effectively a calibration curve for the CMF sensor, which is used in later experiments involving the detection of PlGF in clinical samples. The corresponding average wavelength shifts at PlGF solution with concentrations of 1 pg/mL, 5 pg/mL, 10 pg/mL, 50 pg/mL,

100 pg/mL, 500 pg/mL, and 1 ng/mL were 0.85, 1.53, 2.42, 3.49, 4.54, 5.77, and 7.24 nm, respectively. The linear fitting result is $y= 2.00x+ 0.47$, with a correlation coefficient of 0.973.

The LoD of the CMF biosensor can be calculated using the following function [14]

$$LoD = 3.3 \times \frac{2.303 S_y x}{k}, \tag{4}$$

where k is the slope of the linear fitting line ($k= 2.00$ in our case), x and S_y are the values and standard deviations of the smallest concentration of the PlGF detected in the experiment (1 pg/mL and 0.13), respectively. Based on **Equation (4)**, the calculated LoD of the CMF biosensor is 0.49 pg/mL.

Comparison of Detection Performance of Different PlGF Sensors

Compared with electrical sensors, the dynamic range of the proposed CMF can better meet the needs of clinical detection. Compared with electrochemical sensors and microfiber Bragg grating, CMF has a lower LoD. Compared with ELISA, CMF has a lower LoD, a shorter detection time, a larger dynamic range, and a lower experimental cost.

Table S1. Comparison of the results for the proposed CMF biosensor with other reported works and commercial testing methods.

Refs.	Method	Testing time (minutes)	Dynamic range (pg/mL)	LoD (pg/mL)	Actual sample testing	Sample size (Test group + control group)
15	Electrical	40	27.6 - 501.6	0.06	√	4 + NA
16	Electrochemical	NA	1 - 2500	53	NA	NA
17	Microfiber Bragg grating	30	5 - 120	5	√	8 + 8
18	Surface plasmon resonance	30	1 – 1000	1.354	√	3 + 7
19	Anion-exchange membrane platform	60	800 – 1600	1000	NA	NA
MULTI SCIENCES® [20]	ELISA	NA	15.63 - 1000	NA	NA	NA
ThermoFisher® [21]	ELISA	80	1.372 - 1000	NA	NA	NA
Sigma® [22]	ELISA	NA	1.372 -1000	NA	NA	NA
This work	CMF biosensor	20	1 - 1000	0.49	√	14 + 21

The Detection Process of ELISA

(i) Each well of the enzyme plate was filled with 300 μL of 1× wash solution and allowed to stand for 30 seconds. After removing the solution, the microtiter plate was inverted and patted dry on lint-free absorbent paper.

(ii) A 100 μL aliquot of 2-fold diluted standard solution was added to the designated standard wells, while blank wells received 100 μL of 5-fold diluted serum.

(iii) For sample wells, 80 μL of 1× assay buffer was dispensed first, followed by 20 μL of serum sample.

(iv) Then, 50 μL of "Detection Antibody Working Solution" was added to each test well.

(v) The plate was sealed with an adhesive sealing membrane and incubated for 2 hours at room temperature.

(vi) After incubation, the liquid was discarded from all wells, and the plate was washed six times with 300 μL of wash solution per well per wash.

(vii) Next, 100 μL of "Streptavidin Working Solution" was introduced into each well.

(viii) The plate was resealed and incubated at room temperature for 45 minutes, after which the washing procedure described in step (vi) was repeated.

(ix) Subsequently, 100 μL of chromogenic substrate was added to each well, and the plate was protected from light and incubated at room temperature for 5–30 minutes.

(x) Finally, the reaction was terminated by adding 100 μL of stop solution to each well. The optical density (OD) was measured using a microplate reader at a maximum absorption wavelength of 450 nm

and a reference wavelength of 570 nm or 630 nm. The calibrated OD value was obtained by subtracting the reference wavelength measurement from the 450 nm measurement.

The Diagnostic Basis of PE and the Classification of Clinical Samples

According to the 2019 World Health Organization and 2020 American College of Obstetricians and Gynecologists guidelines [23, 24], the pre-eclampsia (PE) patients diagnosed by sustained elevated blood pressure ≥ 140/90 mmHg or accompanied by random urine protein 2+ or urine protein/creatinine ratio ≥ 300 mg/g or other end organ damage, or fetal demise, or deterioration of the fetal status (new occurrence of fetal doppler abnormality, deterioration of fetal doppler indices, and deterioration of cardiotocographic indices, etc.). The control group included only gestational diabetes mellitus (GDM) and healthy pregnant women. We analyzed serum samples from a group of PE patients (n= 14, gestational range of 33 to 39 weeks, Group 1), hypertension in pregnancy in non-PE patients (n= 5, gestational range of 35 to 39 weeks, Group 2), only GDM (n= 9, gestational range of 37 to 40 weeks, Group 3), and healthy pregnant women (n= 7, gestational range of 37 to 41 weeks, Group 4).

Table S2 shows the baseline characteristics collected for all the pregnant women included in the study. We separately recorded the age, height, weight, gravida, and para. For all analyzed characteristics, there were no significant differences in P values between group 1 and the other groups (P> 0.015), which means that PlGF can be analyzed as a relatively independent indicator.

Table S2. Baseline characteristics of all surveyed pregnant women.

Variable	PE (n= 14)	Hypertension in pregnancy in non-PE (n= 5)	Only GDM (n= 9)	Healthy control (n= 7)
Age	Mean= 31.5<br>SD= 3.8	Mean= 31.8<br>SD= 1.1	Mean= 30.3<br>SD= 2.0	Mean= 29.1<br>SD= 3.3

Height (cm)	Mean= 159.9<br>SD= 5.2	Mean= 156.2<br>SD= 2.4	Mean= 159.4<br>SD= 6.3	Mean= 159.9<br>SD= 4.6
Weight (kg)	Mean= 73.9<br>SD= 10.9	Mean= 66.5<br>SD= 10.9	Mean= 71.3<br>SD= 11.0	Mean= 63.3<br>SD= 6.3
Gravida	Mean= 2.4<br>SD= 1.1	Mean= 1.2<br>SD= 0.4	Mean= 2.8<br>SD= 1.5	Mean= 2.2<br>SD= 1.2
Para	Mean= 0.6<br>SD= 0.6	Mean= 0.2<br>SD= 0.4	Mean= 1.3<br>SD= 1.1	Mean= 0.9<br>SD= 0.8

The Auxiliary Diagnosis Value of PlGF

The percentage of patients presenting with each of the symptoms in relation to the total number of patients with PE was 7.1%, 21.4%, 50%, 28.6%, 35.7%, 14.3%, 7.1%, 7.1%, 35.7% and 92.9%, respectively. The percentage of hypertensive patients during pregnancy was 5.3%, 31.6%, 52.6%, 31.6%, 26.3%, 15.8%, 5.3%, 5.3%, 26.3%, and 68.4%, respectively, as shown in **Figure 4(a)**.

Among patients with PE, the proportions of patients presenting with the aforementioned symptoms were: 7.1%, 21.4%, 42.9%, 7.1%, 21.4%, 21.4%, 14.3%, 0%, 28.6%, and 71.4%, as shown in **Figure 4(b)**. As illustrated in **Figure 4(b)**, these percentages of patients screened utilizing the low PlGF level method were 10%, 20%, 60, 0%, 30%, 20%, 20%, 0%, 30%, and 80%, respectively.

As shown in **Figure 4(c)**, the sensitivity, specificity, and accuracy of using the low levels of PlGF in patients with gestational hypertension to predict PE were 0.5, 0.8, and 0.58, respectively. In contrast, the sensitivity, specificity, and accuracy of using the cut-off values obtained from the CMF test to predict PE were 0.85, 0.5, and 0.74, respectively. The sensitivity and accuracy by the CMF cut-off value method is better than those of the low PlGF level method. The specificity of the low PlGF level method is better than CMF cut-off value method. **Figure 4(d)** shows the screening effect of the low

PlGF level method and the CMF cut-off value method on all samples. As shown in **Figure 4(d)**, the sensitivity, specificity, and accuracy of using the low levels of PlGF in all collected patients to predict PE were 0.5, 0.95, and 0.77, respectively. In contrast, the sensitivity, specificity, and accuracy of using the cut-off values obtained from the CMF test to predict PE were 0.79, 0.86, and 0.83, respectively.

REFERENCES

[1] Tian, Z.; Xuan, H.; Yao, Y.; Hao, S.; Zhang, Z.; Zhang, B.; Zhang, J.; Zhang, L.; Sang, X.; Yuan, J.; Farrell, G.; Wu, Q. Fast Detection of Protein Kinase B in Chrysin Treated Colorectal Cancer Cells Using a Novel Multicore Microfiber Biosensor. *Communications Engineering* **2024**, *3*(1), 185.

[2] Zhang, Y.; Yang, P.; Wei, W.; Jiang, S.; Bao, W.; Zhu, X.; Wu, G.; Chen, D. Highly Sensitive Salinity Sensor Based on Virtual Vernier Effect of Micro–Nano Fiber Mode Interferometer. *IEEE Sensors Journal* **2024**, *24*(10), 16121–16129.

[3] Zhao, Y.; Li, C.; Lin, Z.; Wang, Y.; Tong, R.; Cai, L. Plug-and-Play Fabry-Perot Interferometric Biosensor with Vernier Effect for Label-Free Detection of Bovine Serum Albumin. *Sensors and Actuators B: Chemical* **2024**, *416*, 135999.

[4] Chen, W.; Wang, J.; Liu, Y.; Geng, T.; Lu, C.; Zeng, X.; Hao, J.; Zhou, J.; Yan, Y.; Yan, Q.; Sun, W. Study on Vernier Effect of Orthogonal Mach-Zehnder Interferometers Based on Polarization-Maintaining Fiber. *Optics & Laser Technology* **2023**, *165*, 109599.

[5] Wang, R.; Yan, M.; Jiang, M.; Li, Y.; Kang, X.; Hu, M.; Liu, B.; He, Z.; Kong, D. Label-Free and Selective Cholesterol Detection Based on Multilayer Functional Structure Coated Fiber Fabry-Perot Interferometer Probe. *Analytica Chimica Acta* **2023**, *1252*, 341051.

[6] Xiong, K.; Fan, Q.; Wu, T.; Shi, H.; Chen, L.; Yan, M. Enhanced Bovine Serum Albumin Absorption on the N-Hydroxysuccinimide Activated Graphene Oxide and Its Corresponding Cell Affinity. *Materials Science and Engineering: C* **2017**, *81*, 386-392.

[7] Li, Z.; Sun, G.; Xie, R. Graphene oxide integrated polarization maintaining microfiber refractometer based on effective birefringence response. *Optics & Laser Technology* **2023**, *164*, 109462.

[8] Yan, S.; Pu, S.; Zhang, Y.; Yuan, M.; Zhang, C. Sensing Properties of Grapheme-Oxide-Functionalized Single-Mode-No-Core-Single-Mode FiberStructure. *Results in Physics* **2021**, *25*, 104310.

[9] Xiao. G.; Zhang, K.; Yang, Y.; Yang, H.; Guo, L.; Li, J.; Yuan, L.; Graphene Oxide Sensitized No-Core Fiber Step-Index Distribution Sucrose Sensor. *Photonics* **2020**, *7*(4), 7040101.

[10] Silva, P.; Romao-Veiga, M.; Ribeiro-Vasques, V.; Peracoli, J.; Peracoli, M.; Amaral, L. Progesterone modulates TNF receptors expression by Jurkat cells cultured with plasma from pregnant women with preeclampsia. *International Immunopharmacology* **2024**, *130*, 111701.

[11] Ampey, A.; Dahn, R.; Grummer, M.; Bird, I. Differential control of uterine artery endothelial monolayer integrity by TNF and VEGF is achieved through multiple mechanisms operating inside and outside the cell – Relevance to preeclampsia. *Molecular and Cellular Endocrinology* **2021**, *534*, 111368.

[12] Song, Y.; Tang, C.; Yin, C. Combination antitumor immunotherapy with VEGF and PlGF siRNA via systemic delivery of multi-functionalized nanoparticles to tumor-associated macrophages and breast cancer cells. *Biomaterials* **2018**, *185*, 117-132.

[13] Catanzaro, J.; Guerriero, J.; Ullman, E.; Sheshadri, N.; Chen, J.; Zong, W.; Elevated Expression of Squamous Cell Carcinoma Antigen (SCCA) Is Associated with Human Breast Carcinoma. *PLOS ONE* **2011**, *6*(4), e19096.

[14] Liu, J.; Liu, B.; Liu, J.; He, X.-D.; Yuan, J.; Ghassemlooy, Z.; Torun, H.; Fu, Y.-Q.; Dai, X.; Ng, W. P.; Binns, R.; Wu, Q. Integrated Label-Free Erbium-Doped Fiber Laser Biosensing System for Detection of Single Cell Staphylococcus Aureus. *Talanta* **2023**, *257*, 124385.

[15] Pham, T. T. T.; Tran, D. P.; Nguyen, M. C.; Amen, M. T.; Winter, M.; Whitehead, C.; Toh, J.; Thierry, B. A Simplified Point-of-Care Testing Approach for Preeclampsia Blood Biomarkers Based on Nanoscale Field Effect Transistors. *Nanoscale* **2021**, *13*(28), 12279–12287.

[16] Soman, S.; Ramakrishnan, K.; Warrier, T. S. Enhancing Pregnancy Care: Harnessing Label-Free Immunosensors for Pre-Eclampsia Detection Using PdNPs/Poly(3,5-Diaminobenzoic Acid) Modified Glassy Carbon Electrodes. *Chemistry Select* **2024**, *9*(37), e202401495.

[17] Cao, S.; Chen, R.; Yang, Q.; He, X.; Chiavaioli, F.; Ran, Y.; Guan, B.-O. Point-of-Care Diagnosis of Pre-Eclampsia Based on Microfiber Bragg Grating Biosensor. *Biosensors and Bioelectronics* **2024**, *249*, 116014.

[18] Huang, Y.; Chen, Y.; Yuan, P.; Luo, B.; Wu, S.; Shi, S.; Zhao, M. Ultra-Sensitive SPR Fiber-Optic Biosensor Based on MNM Structure with Ti_3C_2 MXene/PDA Modification for Placental Growth Factor Detection. *Sensors and Actuators B: Chemical* **2025**, *443*, 138214.

[19] Ramshani, Z.; Fan, F.; Wei, A.; Romanello-Giroud-Joaquim, M.; Gil, C.-H.; George, M.; Yoder, M. C.; Hanjaya-Putra, D.; Senapati, S.; Chang, H.-C. A Multiplexed Immuno-Sensor for on-Line and Automated Monitoring of Tissue Culture Protein Biomarkers. *Talanta* **2021**, *225*, 122021.

[20] MULTI SCIENCES® Human PlGF ELISA Kit, https://www.liankebio.com/product/human-pigf-elisa-kit-6110.html.

[21] Thermo Fisher® Human PlGF-1 ELISA Development Kit (ABTS), https://www.thermofisher.cn/cn/en/elisa/product/Human-PlGF-1-ELISA-Development-Kit-ABTS/900-K307K.

[22] Sigma® Human Placental Growth Factor ELISA Kit, https://www.sigmaaldrich.cn/CN/en/product/sigma/rab0404.

[23] World Health Organizations, Pre-eclampsia, https://www.who.int/news-room/fact-sheets/detail/pre-eclampsia.

[24] The American College of Obstetricians and Gynecologists. *222*, https://www.preeclampsia.org/frontend/assets/img/advocacy_resource/Gestational_Hypertension_and_Preeclampsia_ACOG_Practice_Bulletin,_Number_222_1605448006.pdf.
